# Dancing molecules: group A bZIPs and PEBPs at the heart of plant development and stress responses

**DOI:** 10.1093/jxb/eraf034

**Published:** 2025-01-26

**Authors:** Damiano Martignago, Vítor da Silveira Falavigna, George Coupland, Lucio Conti

**Affiliations:** Dipartimento di Bioscienze, Università degli Studi di Milano, Milan, Italy; Max Planck Institute for Plant Breeding Research, Cologne, Germany; Max Planck Institute for Plant Breeding Research, Cologne, Germany; Dipartimento di Bioscienze, Università degli Studi di Milano, Milan, Italy; University College Dublin, Ireland

**Keywords:** ABA, bZIPs, development, FD, FLORIGEN ACTIVATION COMPLEX, flowering, group A bZIPs, PEBPs, seed germination

## Abstract

Group A basic leucine zipper (bZIP) transcription factors play critical roles in abscisic acid (ABA) signaling and plant development. In *Arabidopsis thaliana*, these factors are defined by a highly conserved core bZIP domain, and four conserved domains throughout their length: three at the N-terminus (C1–C3) and a phosphorylatable C-terminal SAP motif located at the C4 domain. Initially, members such as ABI5 and ABFs were studied for their roles in ABA signaling during seed germination or stress responses. Later, a sub-clade of group A bZIPs, including FD, was found to play important roles in floral induction by interacting with the florigen FLOWERING LOCUS T (FT) at the shoot apical meristem. Recent research has expanded our understanding of these transcription factors by identifying intriguing parallels between those involved in ABA signaling and those promoting floral induction, and revealing dynamic interactions with FT and other phosphatidylethanolamine-binding proteins (PEBPs) such as TERMINAL FLOWER 1. Studies in crop plants and non-model species demonstrate broader roles, functions, and molecular targets of group A bZIPs. This review highlights common features of group A bZIPs and their post-translational regulation in enabling the activation of gene regulatory networks with important functions in plant development and stress responses.

## Teaser

We discuss recent advances in understanding group A bZIP transcription factors from Arabidopsis and crops, and their interactions with FT and other florigen-like PEBPs in ABA signaling, seed germination, and floral transitions

## Introduction

**Fig. 1.**
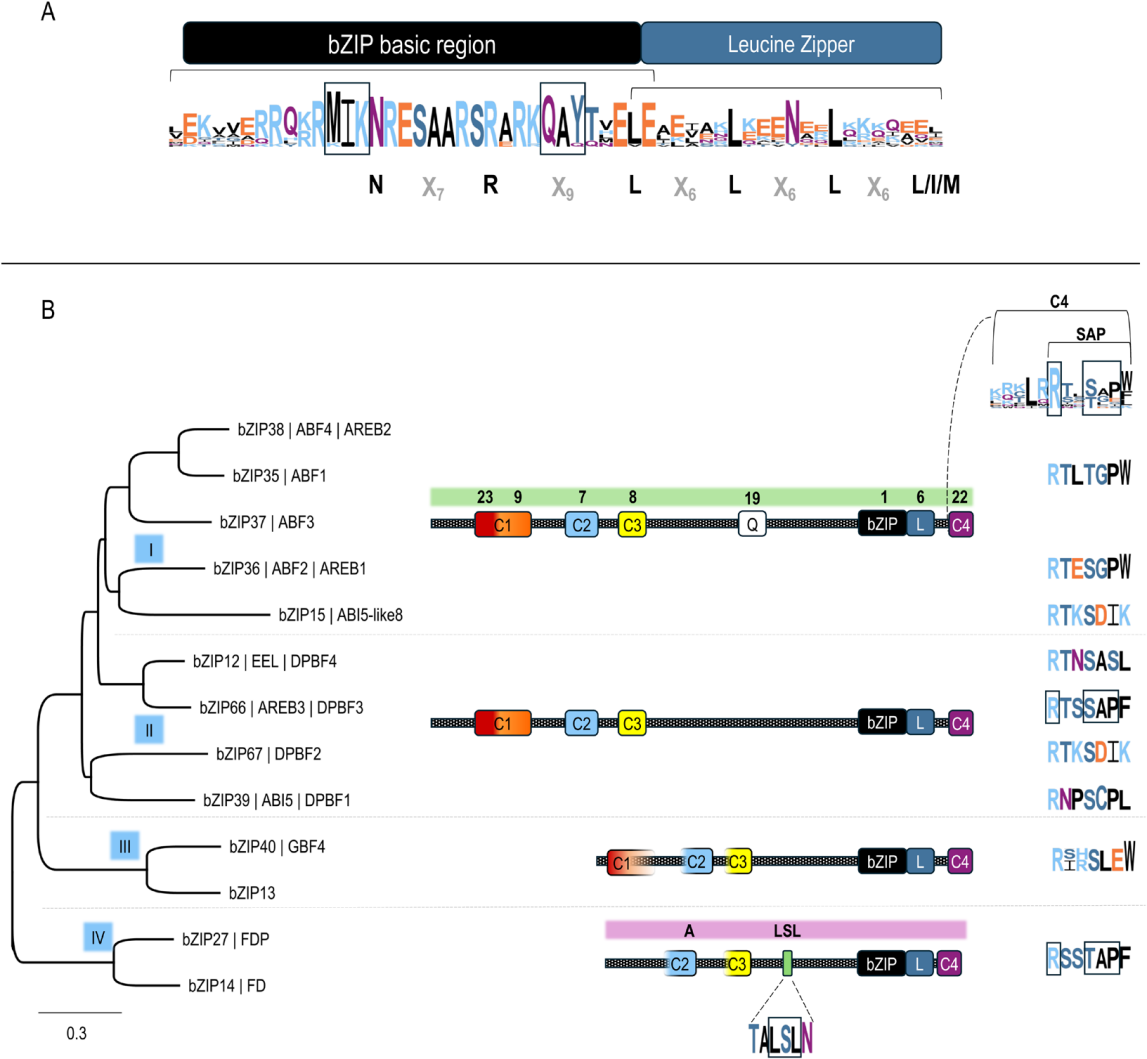
Functional classification of Arabidopsis group A bZIPs (Jakoby *et al.*, 2002; Tsuji *et al.*, 2013; Dröge-Laser *et al.*, 2018). (A) Protein logo of the bZIP domain. The unique M/K-I-K and Q-A-Y/Q residues are boxed (Bensmihen *et al.*, 2002; Jakoby *et al.*, 2002). With the exception of subgroup II AREB3, EEL, and DPBF2, all the other proteins have an extra -x_6_-L/I/M leucine repeat at the C-terminus of the bZIP domain. Protein sequence logos were designed with WebLogo 3 (Crooks *et al.*, 2004). (B) A phylogenetic Neighbor–Joining tree was produced with Geneious Prime Tree Builder (Blosum 80 align) using the whole protein sequences, with GBF3 as an outgroup. Blue boxes within the phylogenetic tree: subgroups I–IV (Bensmihen *et al.*, 2002). Dotted boxes represent the protein scheme. The color boxes on top of the protein scheme identify the conserved motifs C1–C4 (Bensmihen *et al.*, 2002), the central bZIP-leucine zipper domain, and, for subgroup I (plus EEL from subgroup II), the polyQ motif (Dröge-Laser *et al.*, 2018). Open boxes represent C1–C3 domains of subgroup III and IV to reflect their partial conservation. Above the protein diagram, the numbered motifs highlighted in lime green correspond to those identified by Dröge-Laser *et al.* (2018). Above the subgroup IV protein scheme, highlighted in magenta, are the A motif (corresponding to the C-terminal region of the C2 motif) and the LSL motif (Tsuji *et al.*, 2013; Dutta *et al.*, 2021). This might serve as disambiguation in nomenclature. The C-terminal C4 motif comprises the later identified SAP motif (Taoka *et al.*, 2011).

In the model species *Arabidopsis thaliana* (Arabidopsis), 78 basic leucine zipper (bZIP) transcription factors (TFs) have been classified into 13 different groups (A–K, plus M and S) based on homology of the bZIP domain and other conserved motifs (Jakoby *et al.*, 2002; Dröge-Laser *et al.*, 2018). The group A bZIP subfamily of Arabidopsis consists of 13 TFs playing roles in abscisic acid (ABA) signaling, germination, and flowering. Structurally, the eponymous bZIP domain contains an N-terminal basic region comprising the DNA-binding site followed by the C-terminal leucine zipper dimerization domain (Jakoby *et al.*, 2002). In group A bZIPs, the basic region is highly conserved and contains two unique M/K-I-K and Q-A-Y/Q motifs (Fig. 1A). These motifs also identify group A bZIPs in other plant species (Tylewicz *et al.*, 2015; Parajuli *et al.*, 2024; Yin *et al.*, 2024). In addition, there are four highly conserved domains named C1–C4 (Fig. 1B). Phosphorylation motifs present within these domains suggest dynamic post-translational regulatory mechanisms (Bensmihen *et al.*, 2002).

Phosphorylation of the C1–C3 N-terminal domains occurs in response to ABA in the group A bZIPs involved in ABA signaling (Furihata *et al.*, 2006; Umezawa *et al.*, 2013; Y. Wang *et al.*, 2013). The phosphorylation site of the C4 domain, located at the very C-terminus of the protein, is present in all Arabidopsis group A bZIPs. In seven of these proteins, the C4 domain presents a conserved sequence called the SAP motif (L-x-R-x-x-S/T-A/G-P, with x representing any amino acid; Fig. 1B), whereas the remaining six have a divergent but functionally similar sequence. SAP motif integrity and its capacity to be phosphorylated at the serine (S) or threonine (T) residues are key for the participation of the bZIPs in multi-protein complexes, and this motif is conserved in group A bZIPs from monocotyledonous (Taoka *et al.*, 2011) and dicotyledonous (Park *et al.*, 2014; Collani *et al.*, 2019) plant species.

**Fig. 2.**
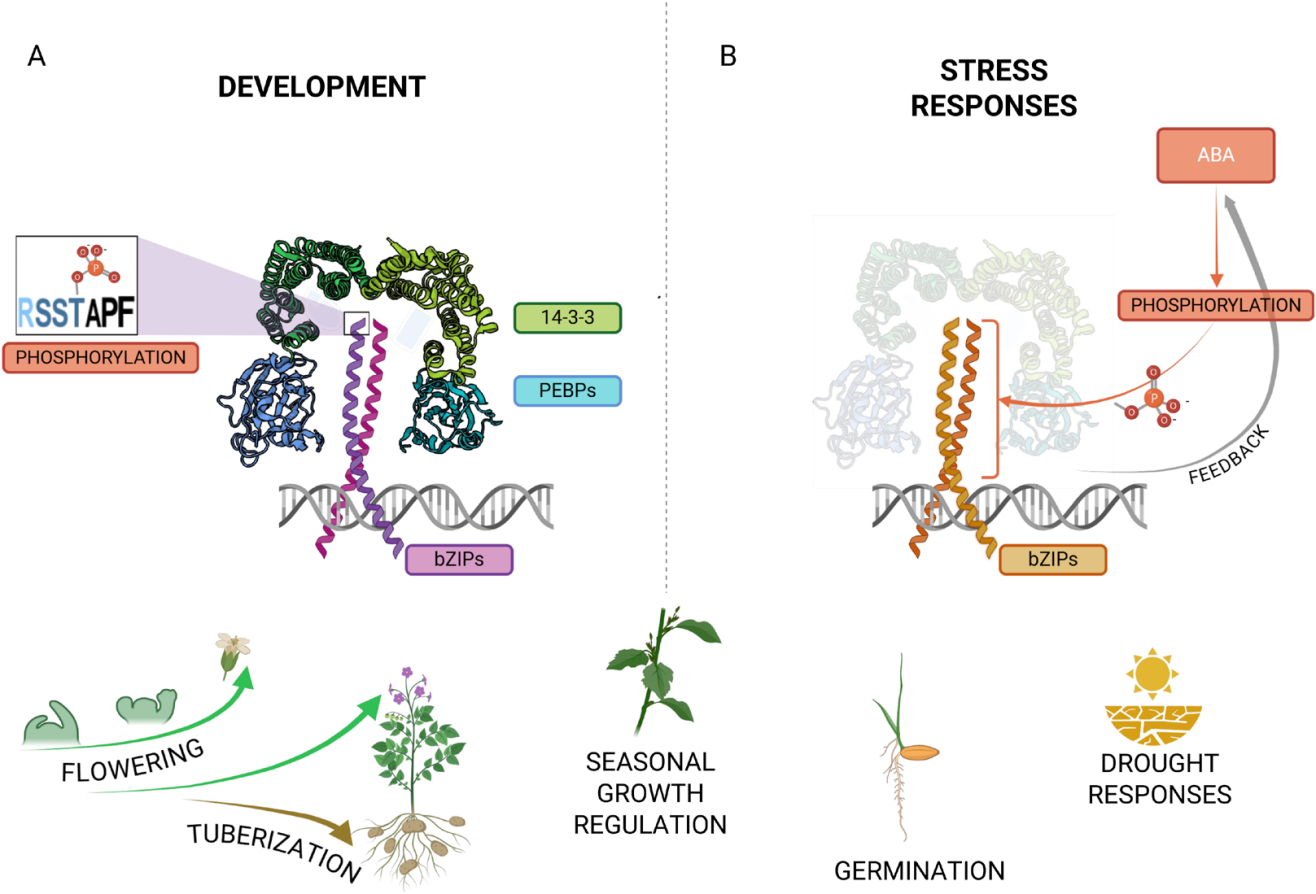
Group A bZIP transcription factors and PEBPs in plant development and stress. (A) Representation of group A bZIP roles and function in FAC-like complexes in developmental processes. SAP phosphorylation requirement for 14-3-3 interaction is highlighted in the box. (B) Representation of group A bZIP functions in stress and ABA responses. As the involvement of a complete FAC-like complex is still under debate, it has been represented in faded colors. Phosphorylation in these processes happens at different S/T residues distributed along the whole protein sequence (orange bracket). Created in BioRender. Martignago D. (2024) https://BioRender.com/f36f242.

A study of the role of the group A bZIP OsFD in floral transition of rice proposed that it functioned within a hexameric complex named the florigen activation complex (FAC). In this complex, a homodimer of the group A bZIP OsFD1 is assembled with a homodimer of the 14-3-3 protein GF14b and two copies of the rice florigen Heading date 3a (Hd3a), such that 14-3-3 interacts with the rice florigen Hd3a and OsFD1 simultaneously and independently (Fig. 2A; Taoka *et al.*, 2011, 2013). OsFD1 is the rice bZIP homolog of the Arabidopsis FD, identified as the molecular partner of the florigen phosphatidylethanolamine-binding protein (PEBP) FLOWERING LOCUS T (FT) (Abe *et al*., 2005; Wigge *et al*., 2005), of which Hd3a is the closest rice homolog. OsFD1 binds directly to DNA on a GACGTC C-box element found in the Arabidopsis promoter of the floral identity gene *APETALA1* (*AP1*) and its homolog *OsMADS15* (Taoka *et al.*, 2011; Collani *et al.*, 2019). This result is consistent with bZIPs binding DNA as dimers, targeting sequences with an ACGT core, flanked by different residues that specify different bZIP target preferences (Hurst, 1995). For group A bZIPs, the G-box (CACGTG), C-box, and the ABA RESPONSIVE ELEMENT (ABRE, ACGTGT/GC) are the most represented *cis*-elements detected *in vivo* (Izawa *et al*., 1993; Menkens *et al*., 1995; Song *et al*., 2016; Collani *et al*., 2019; Romera-Branchat *et al*., 2020; Zhu *et al*., 2020).

Here, we summarize the role of group A bZIP TFs in plant development, tissue and organ differentiation, and stress responses. We examine their conservation and their biological functions across various plant species, emphasizing key specializations. Additionally, we explore reports of the formation of FAC or FAC-like complexes in different contexts. We discuss the current understanding of bZIP homo- and heterodimerization, as well as post-translational modifications—particularly phosphorylation—and how these factors affect bZIP activity or their DNA binding properties. We will explore recent advances in understanding Arabidopsis group A bZIP TFs, and the main insight from the study of their homologs in crops and non-model species.

## Functional classification of group A bZIP transcription factors

Group A bZIP TFs have been classified into four subgroups, named I–IV (Fig. 1B) (Bensmihen *et al.*, 2002). Subgroup I comprises the four ABA-RESPONSIVE ELEMENT BINDING FACTORs (ABF1–ABF4, also known as AtbZIP35, 36, 37, and 38, respectively), plus the uncharacterized bZIP15. Subgroup II includes ABA INSENSITIVE 5 (ABI5, otherwise known as Dc3 promoter-binding factor 1, DPBF1, AtbZIP39), DPBF2 (AtbZIP67), and the two highly similar ABA-RESPONSIVE ELEMENT BINDING PROTEIN 3 (AREB3, also known as DPBF3, AtbZIP66) and ENHANCED EM LEVEL (EEL, also known as DPBF4, AtbZIP12). In monocot studies, this subgroup is sometimes further divided into an ABI5 subfamily (ABI5 and DPBF2 homologs) and a DBPF subfamily (AREB3 and EEL homologs) (Tsuji *et al.*, 2013; Zhou *et al.*, 2017; Utsugi *et al.*, 2020). Subgroups I and II contain conserved C1–C4 domains (Bensmihen *et al.*, 2002). In addition, subgroup I and EEL from subgroup II have a polyglutamine (polyQ) repeat (Dröge-Laser *et al.*, 2018) that is associated with protein–protein interactions (Barbosa Pereira *et al.*, 2023) and might confer thermal responsiveness to the protein structure (Jung *et al.*, 2020). Subgroup III contains the uncharacterized proteins bZIP13 and G-BOX-BINDING FACTOR 4 (GBF4, also known as AtbZIP40). In members of subgroup III, the C1 domain is poorly conserved, while C2 and C3 are conserved only in the C-terminal part. The C4 domain has a divergent L-x-R-x-x-S-L-E-W motif instead of the canonical SAP motif (Fig. 1B). Subgroup IV contains the flowering time regulators FD (AtbZIP14) and FDP (FD PARALOG, also known as AtbZIP27). FD and FDP proteins feature a shortened N-terminal region, which lacks the C1 domain and is missing the N-terminal portion of the C2 (referred to as motif A, Fig. 1) and C3 domains. In grasses (*Poaceae*), FD homologs exhibit a unique N-terminal motif, known as Motif 1, in place of the partial C2 domain (Fig. 1B) found in dicots and non-*Poaceae* monocots, such as bananas, palms, and orchids (Tsuji *et al.*, 2013; Dutta *et al.*, 2021). Between the partial C3 and the bZIP domain there is an LSL motif (T-A/V-L-S-L-N, Fig. 1B), which is highly conserved in orthologs from other plant species (Tsuji *et al.*, 2013; Dutta *et al.*, 2021). The C4 domain of ABF1–ABF4, AREB3, and subgroup IV bZIPs contains a functionally characterized SAP motif (R-x-x-S/T-A/G-P/Q, Taoka *et al.*, 2013; Park *et al.*, 2014), with the flowering-related version (R-x-x-S/T-A-P) fully conserved only in subfamily IV and in AREB3, along with their homologs in other species (Tsuji *et al.*, 2013; Utsugi *et al.*, 2020; Kaur *et al*., 2021).

Many phosphorylation events on S/T residues in the C1–C4 domains have been experimentally validated (Furihata *et al.*, 2006). However, phosphorylation can also occur at residues outside of these conserved domains. For example, subgroup III GBF4 lacks a conserved C1 domain with a recognizable phosphorylation consensus, yet phosphorylation at N-terminal serine residues has been experimentally observed (Wang *et al.*, 2018). Similarly, AREB3 and EEL exhibit a conserved SQS15 amino acid triplet preceding the C1 domain, where both the S residues are phosphorylated (Al-Momani *et al.*, 2018).

The functional classification presented here generally reflects the biological roles of the subgroup members, frequently highlighting gene redundancy within each subgroup. Subgroup I ABFs were initially characterized for their redundant role in ABA responses, and subgroup II ABI5 and EEL for their control of embryogenesis and germination (Collin *et al.*, 2021). While subgroup III remains poorly investigated, subgroup IV FD and FDP have long been known to be involved in flowering (Abe *et al*., 2005; Wigge *et al*., 2005; Jaeger *et al*., 2013). However, more recent studies revealed significant redundancy among group A bZIPs in different subclasses. For example, AREB3 assigned to ABA signaling acts partially redundantly with FD and FDP in promoting the floral transition (Martignago *et al.*, 2023), whereas mutants for FD and FDP show reduced ABA sensitivity during seed germination as well as flowering (Martignago *et al.*, 2020; Romera-Branchat *et al.*, 2020).

## Group A bZIPs mediate ABA responses and control seed traits and bud dormancy

Seed germination is the first developmental transition in the growth of flowering plants and is precisely timed by seasonal and environmental cues. Seed germination can be prevented by dormancy, which occurs when environmental conditions are unsuitable for seedling survival. A central endogenous pathway regulating germination involves a balance between ABA and gibberellin (GA), with ABA preventing premature germination in harsh environmental conditions. In addition, other phytohormones such as brassinosteroids, cytokinins, and jasmonate, as well as the circadian clock, stress responses (Collin *et al.*, 2021), and light stimuli converge on the group A bZIP ABI5 to regulate germination (Zhao *et al.*, 2022). The *abi5* mutant was identified because it was able to germinate in the presence of high concentrations of ABA (Finkelstein, 1994; Finkelstein and Lynch, 2000). ABA induces *ABI5* transcription and regulates ABI5 accumulation by preventing its degradation (Lopez-Molina *et al.*, 2001). ABA-activated ABI5 binds to ABRE sequences in its target genes (Carles *et al.*, 2002), ultimately arresting seedling growth in unfavorable conditions (Lopez-Molina *et al*., 2001, 2002; Xi *et al*., 2010). ABI5 is subject to different types of post-translational modifications (which have been extensively reviewed), including ABA-triggered phosphorylation (Lopez-Molina *et al*., 2002; Yu *et al*., 2015), which is required for ABI5 activity and its ability to regulate gene expression (Y. Wang *et al.*, 2013). To control seed dormancy, ABI5 interacts genetically with the PEBP MOTHER OF FT AND TFL1 (MFT) in a negative feedback loop involved in ABA signaling. ABI5 binds to the *MFT* promoter, increasing its transcription in Arabidopsis embryos, which is also promoted by ABA. In turn, MFT represses ABI5 expression and ABA signaling in a pathway which has yet to be fully elucidated (Xi *et al.*, 2010), involving GA, light signals, and circadian clock genes (Xi *et al.*, 2010; Footitt *et al.*, 2017; Vaistij *et al.*, 2018). In the early stages of seed development, ABI5 interacts with a second PEBP, TERMINAL FLOWER 1 (TFL1), which stabilizes ABI5 and regulates endosperm cellularization (Zhang *et al*., 2020). In rice, the ABA-inducible ABI5 homolog OsABI5/OREB1/OsbZIP10 (Zou *et al.*, 2008) forms a FAC-like complex with the MFT homolog MFT2 and the monocot-specific 14-3-3 protein GF14h (Yoshida *et al*., 2022). The transcriptional activity of OsABI5 depends on the recruitment of GF14h–OsMFT2 to the nucleus to form a trimeric complex that attenuates seed germination, whereas GF14h suppresses ABA signaling by reducing OsABI5 transcriptional activity in the absence of OsMFT2 (Yoshida *et al*., 2022). Other subgroup I rice homologs, such as OsbZIP23, OsbZIP66/TRAB1, and OsbZIP72, also interacted with OsMFT2, highlighting redundancy in ABA-dependent repression of seed germination in rice (Song *et al.*, 2020). In wheat, at least three ABI5 homologs are expressed in seeds or seedlings: TaABF1 (Johnson *et al.*, 2002), the drought- and ABA-inducible Wabi5 (Kobayashi *et al.*, 2008), and TaABI5 (Zhou *et al.*, 2017), with the latter increasing ABA sensitivity and inhibiting germination when heterologously overexpressed in Arabidopsis (Utsugi *et al.*, 2020). A wheat seed-specific MFT homolog is involved in seed dormancy and germination, but the association with bZIPs was not investigated (Nakamura *et al.*, 2011). These observations suggest that FAC-like complexes formed by ABI5 and PEBPs have important roles in seed germination.

Similar to seed dormancy, bud dormancy—an adaptive process that allows perennial trees to survive harsh winter conditions—is antagonistically regulated by the hormones ABA and GA (Ding *et al.*, 2024; Sato and Yamane, 2024; Zhao and Wang, 2024). The levels of ABA roughly correlate with the different phases of bud dormancy, with dormancy-inducing conditions triggering the accumulation of ABA, followed by a decline towards the release of dormancy. As expected, the expression and function of genes related to ABA biosynthesis and signaling were also found to be differentially regulated during bud dormancy in many perennial species. A complex feedback circuit occurs between ABA–ABFs and the central bud dormancy regulators called DORMANCY-ASSOCIATED MADS-BOX (DAM) (Sato and Yamane, 2024; Falavigna *et al.*, 2019). Several ABF-like TFs have been proposed to regulate the expression of *DAM* genes during different dormancy phases. In pear, PpyABF3 induces *PpyDAM3* and *PpyDAM4* expression after dormancy establishment, whereas PpyABF2 can repress *PpyDAM1* close to budbreak (Tuan *et al.*, 2017; Yang *et al.*, 2020, 2023). In addition, a heterodimer between PpyABF2 and PpyABF3 is formed at the end of bud dormancy to inhibit PpyABF3 binding to the *PpyDAM3* promoter (Yang *et al.*, 2020). The peach homolog of PpyABF2 also regulates the expression of *DAM* genes (Wang *et al.*, 2020). Outside subgroup I bZIPs, FDL proteins regulate dormancy in response to photoperiod. In aspen, FDL1 has distinct functions, with a complex consisting of FT and FDL1 mediating photoperiodic control of seasonal growth and an FT-independent module controlling adaptive responses through interaction with the homolog of ABI3 (Tylewicz *et al*., 2015; Sheng *et al.*, 2022).

In Arabidopsis, several group A bZIPs also mediate ABA responses in vegetative tissues. Quadruple mutants of *ABF1–ABF4* show increased sensitivity to water deficit as a result of impaired expression of ABA-activated transcripts, many involved in osmotic stress response and tolerance (Yoshida *et al.*, 2010, 2015). *ABF3* mRNA peaks in the morning but also shows another peak of induction by ABA at midday. This pattern is regulated by the circadian clock, suggesting a tight diel regulation of ABA transcriptional responses mediated by ABF3 (Liang *et al.*, 2024). In addition, EEL regulates the diurnal transcriptional activation of *9-cis-epoxycarotenoid dioxygenases* 3 (*NCED3*), encoding a rate-limiting step in ABA biosynthesis. EEL binds to an ABRE *cis-*element in the promoter of *NCED3* to increase its transcription (Baek *et al.*, 2020). Notably, EEL also physically associates with GIGANTEA (GI), which is a plant-specific circadian clock-regulated protein involved in multiple environmental pathways, and GI shows binding at the *NCED3* promoter, favoring its activation (Baek *et al.*, 2020). While the precise role of GI in this transcriptional mechanism is still unclear, ChIP-seq meta-analyses indicate that GI and several ABFs significantly co-localize at promoters of genes that are regulated by ABA and water deficit (Siemiatkowska *et al.*, 2022), suggesting that GI might be an additional factor regulating bZIP functions at specific promoters. Thus, the accumulation pattern of different bZIPs could provide plants with ABA transcriptional responsiveness according to diel variation in water availability.

Plants primarily lose water through transpiration, a process regulated by guard cell movements that control the opening and closing of stomata. ABA-induced stomatal closure is driven by the osmotic expansion of guard cells, which results from the accumulation of K^+^ ions transported by blue-light-responsive membrane H^+^-ATPases (Kinoshita and Shimazaki, 1999). Additionally, an ABA-independent, photoperiod-dependent module involving FT, 14-3-3 proteins, and H^+^-ATPases also plays a role in regulating stomatal movement (Kinoshita *et al.*, 2011). Other photoperiodic genes acting upstream of *FT*, notably *GI* and *CONSTANS*, are also involved in stomatal regulation (Ando *et al.*, 2013). Expression of *FD*, *TFL1*, and *TWIN SISTER OF FT* (*TSF*) has been detected in guard cells, leading to the hypothesis that FT induces stomatal opening via a pathway similar to floral induction (Kinoshita *et al.*, 2011). Supporting this proposal, expression of several floral targets of FT/FD normally activated at the shoot apex was detected in guard cells (Kinoshita *et al.*, 2011; Kimura *et al.*, 2015; Aoki *et al.*, 2019). However, the role of the FT/FD complex in regulating stomatal movement is still unclear. Beyond FD, subgroup I bZIPs (ABF1–ABF4) and subgroup II AREB3 and EEL are also expressed in leaves (Qian *et al.*, 2019). However, no alterations in stomatal opening were observed in *abf2-3-4* triple mutants, *abf1–abf4* quadruple mutants (Yoshida *et al.*, 2015), or *ABF2*-overexpressing plants (Fujita *et al.*, 2005). AREB3 promotes the expression of *Actin-depolymerizing factor 5* (*ADF5*), a cytoskeleton remodeling factor whose mutants have impaired ABA-induced stomatal closure (Qian *et al.*, 2019). Still, single *areb3-1* and double *areb3-1 eel* mutants do not present this phenotype, possibly due to redundancy with other group A bZIPs that can also regulate *ADF5* (Qian *et al.*, 2019), or because of the residual expression of the *areb3-1* insertional mutant (Qian *et al.*, 2019; Martignago *et al.*, 2023). Interestingly, *eel* mutants display increased stomatal aperture under water deficit conditions, suggesting that the contribution of bZIP TFs to regulate stomatal movement could be stress specific (Baek *et al.*, 2020).

## Molecular insights derived from ABA-regulated bZIPs

Much of our knowledge about the molecular functions of group A bZIPs derives from studying subgroup I and their role in relaying ABA/osmotic stress-dependent transcriptional reprogramming. In seedlings, water deficit/salt stress conditions promote the transcriptional activation of these bZIPs, particularly ABI5, ABF2, ABF3, and ABF4 (Fujita *et al.*, 2005). Importantly, ABA triggers post-transcriptional activation through phosphorylation at several conserved R-x-x-S/T sites in AREB/ABFs by SNF1-related kinase 2 (SnRK2) protein kinases (Uno *et al.*, 2000; Furihata *et al.*, 2006; Fujii *et al.*, 2007). These phosphorylation events have been described independently through MS approaches *in vivo* (Lopez-Molina *et al*., 2002; Furihata *et al.*, 2006; Kline *et al*., 2010; P. Wang et al., 2013; Minkoff *et al*., 2015) and *in vitro* (Furihata *et al.*, 2006; Nakashima *et al.*, 2009; P. Wang *et al.*, 2013).

The significance of phosphorylation on the functions of subgroup I bZIPs was studied in protoplast transactivation assays, using wild-type or mutated versions of ABF2 in combination with a *GUS* (*β-glucuronidase*) reporter gene fused to the 77 bp fragment containing two ABRE motifs (Furihata *et al.*, 2006). Most amino acid substitutions at various phosphorylation sites reduced the expression of the reporter, particularly when the four T/S phosphorylation sites in the C1–C4 regions were replaced with alanine. Conversely, substituting these sites with aspartate, which mimics the phosphorylated state, led to constitutive *GUS* expression, regardless of ABA treatment. These results broadly point to a key role for ABA-mediated phosphorylation in promoting ABF2 function, although phosphorylation events at the C1–C4 domains may control distinct aspects of bZIP functions, including protein stability and transactivation ability.

The stabilization of bZIP proteins has been reported to result from ABA treatment, and to possibly play a major role in recruitment of the bZIP TFs to chromatin (Song *et al.*, 2016; Liang *et al.*, 2024). Protein stabilization is dependent on specific phosphorylation events at the C4 domain. Mutants of ABF1 and ABF3 at their C-terminus (thus depleted of their phosphorylation site located in the C4 domain) are unstable and cannot accumulate to wild-type levels in plant cells (Linden *et al.*, 2021). This result was similar to the reduced stability observed in the yellow fluorescent protein (YFP)–abf3^T451A^ point mutant of ABF3 *in vivo* (Sirichandra *et al.*, 2010). In contrast, the abf3^S126A^ mutant protein, lacking an ABA-regulated phosphorylation site in the N-terminal region, is still phosphorylated at the C4 domain and accumulates in plant nuclei. Moreover, unlike the wild-type YFP–ABF3 protein, the mutant YFP–abf3^T451A^ protein was not stabilized upon ABA treatment. Its detection and immunoprecipitation were only possible after application of MG132, a proteasome inhibitor. This suggests that phosphorylation at the C4 domain is important for the ABA-mediated stabilization of ABF3. Phosphorylation at T451 of ABF3 promotes its interaction with 14-3-3 proteins, which could play a role in stabilizing ABF3 and/or regulating its global function. Accordingly, analysis of the immunoprecipitated mutant version of YFP–abf3^T451A^ did not reveal additional phosphorylation events at other sites in response to ABA. Considering that the abf3^S126A^ mutant is still significantly phosphorylated in response to ABA, one possibility is that phosphorylation of T451 can prime the subsequent phosphorylation of other sites, thus affecting ABF3 function.

It remains unclear whether the role of C4 phosphorylation in mediating ABF1/2/3 protein stability applies to all group A bZIPs, as other regulatory mechanisms may be involved. For instance, the *abi5*^ΔC4^ mutant was degraded more slowly than the full-length ABI5, suggesting that the role of the C-terminal region in protein stabilization is not conserved (Liu and Stone, 2013). Interestingly, the formation of the TFL1–ABI5 complex plays a crucial role in promoting ABI5 stability, particularly in regulating endosperm cellularization (Zhang *et al.*, 2020). Moreover, while generally unstable, overexpression of *abf1*^ΔC4^ could still cause delayed germination. This effect was less pronounced compared with the wild type, suggesting that ABF1 retains partial activity without its C-terminal phosphorylatable residues (Linden *et al.*, 2021). This observation could also explain why the mutant abf2^S413A^ can transactivate the ABRE-regulated reporter in protoplast assays similarly to the wild-type ABF2 (Furihata *et al.*, 2006). Conversely, when expressed under native promoter conditions, AREB3 mutant proteins lacking the SAP motif were not active in promoting flowering (Martignago *et al.*, 2023), supporting a key role for the C4 region—and possibly for the phosphorylatable amino acid encoded in its SAP motif. At least qualitatively, areb3^ΔSAP^ proteins were still detectable in plant nuclei, unlike abf3^T451A^. However, a direct comparison to assess stability would require more investigations.

Protein destabilization is unlikely to account for the reduced transactivation assays of other phosphonull mutants of ABF2 in the N-terminal portion of the proteins (Furihata *et al.*, 2006). Moreover, a deletion of 60 amino acids (referred to as region P) from the N-terminus decreased *GUS* expression with or without exogenous ABA. This region possibly confers transactivation potential to ABF2, a finding also supported by recent efforts aimed to systematically map Arabidopsis TF transactivation domains, including most group A bZIPs (Morffy *et al.*, 2024). Moreover, overexpression of a chimeric version of ABF2 (referred to as areb1^ΔQT^, consisting of the N-terminal transactivation domain and the C-terminal DNA-binding and C4 domains of ABF2) showed constitutive activation of ABF2 targets normally activated by ABA (Fujita *et al.*, 2005). Because the areb1^ΔQT^ phenotype and activity are still enhanced by water deficit and ABA applications, ABA could still influence other aspects of this protein’s functions. While much research has focused on the molecular aspects of the function and stability of bZIPs in relation to DNA binding, the regulation of transcription itself remains less clearly understood. Some insights come from the natural diversity observed in the ABF2 N-terminal domain (Des Marais *et al*., 2015). Among 238 Arabidopsis accessions sampled from diverse geographic regions, two predominant haplotypes of the *ABF2* gene were identified: the reference Col-0 type and the Wassilewskija (Ws) type. The Ws-type allele contains small insertions and polymorphisms causing extra amino acid additions or changes in the N-terminal domain protein sequence which could alter its transcriptional activity. Complementation of *abf2* loss-of-function mutants with either the Col-0- or Ws-derived alleles, followed by RNA-seq analysis, revealed distinct allelic effects on genome-wide expression levels under well-watered but not under water deficit conditions. Thus, the Ws-derived *ABF2* allele could play a role in environmental adaptation, by shaping specific gene expression patterns in water-abundant environments.

## Group A bZIPs involved in flowering and differentiation

Flowering time in plants must align with the most favorable season for reproductive success, which is partly achieved by sensing changes in day length (photoperiod). These changes are detected in the leaves, triggering the production of a systemic signal known as florigen, which induces flowering at the shoot apical meristem (SAM). PEBPs have been identified as the main components of the florigen signal. Florigen signaling occurs in three stages: photoperiodic and environmental regulation of its production in the leaf vasculature (Takagi *et al*., 2023); its transport to the SAM (Liu *et al*., 2020); and its role in regulating gene expression to promote flowering. The first two stages have been extensively reviewed (Liu *et al.*, 2013; Putterill and Varkonyi-Gasic, 2016; Colleoni *et al.*, 2024; Maple *et al.*, 2024). This discussion will focus on the role of group A bZIPs. These proteins, together with PEBPs, initiate the transcriptional changes that trigger floral transition and may also regulate other developmental transitions.

The contribution of FD to florigen signaling was highlighted in multiple studies employing genetics and suppressor screens (Koornneef *et al.*, 1991; Abe *et al.*, 2005) or yeast-based assays for PEBP interactors (Wigge *et al*., 2005). Collectively, these studies in Arabidopsis revealed that FD function was limiting for FT signaling at the shoot apex. In one possible model, the expression of FD at the SAM could offer FT a spatial coordinate for the activation of floral genes (Wigge *et al*., 2005; Abe *et al*., 2019). Indeed, ChIP-seq confirmed FD binding to floral integrators such as *SUPPRESSOR OF OVEREXPRESSION OF CONSTANS 1* (*SOC1*) and *FRUITFULL* (*FUL*), as well as floral meristem identity genes such as *AP1* and *LEAFY* (*LFY*) (Collani *et al.*, 2019; Romera-Branchat *et al.*, 2020; Zhu *et al.*, 2021). Interestingly, FDP seems to be more specialized in binding to genes involved in ABA responses, some of which are also bound by FD. However, while *fdp* single mutants weakly regulate flowering, the pyramiding of *fdp*, *areb3*, and *fd* aggravates the late-flowering phenotype of *fd* single mutants (Jaeger *et al.*, 2013; Romera-Branchat *et al.*, 2020; Martignago *et al.*, 2023). In tomato, *SUPPRESSOR OF SELF-PRUNING* (*SSP*), a homolog of *FD*, is a regulator of flowering and meristem determinacy (Park *et al*., 2014), and its paralog *SSP2* has partially redundant roles (Glaus *et al.*, 2025). Notably, a deleterious mutation in *SSP2* was prevalent in domesticated germplasm, and genome editing was used to repair this mutation, which resulted in desirable traits such as compact growth and early fruit yield (Glaus *et al.*, 2025). However, in rice, no redundancy was observed between *OsFD1* and *OsFD4*, because *osfd1* or *osfd4* single mutants showed delayed flowering, but no further delay in flowering was observed in *osfd1 osfd4* double mutants (Taoka *et al*., 2011; Cerise *et al*., 2021). Nevertheless, OsbZIP65/OsFD7 is an AREB3 homolog that can interact with PEBPs and 14-3-3s, and is also involved in floral transition (Kaur *et al.*, 2021), which suggests an intricate network of group A bZIP TFs capable of transducing florigen signals at the rice SAM. Thus, although different subgroups of group A bZIPs have developed distinct functional and molecular preferences, their selectivity for physiological processes is likely to be more nuanced. In some cases, compensatory expression mechanisms among related bZIPs may enhance the activation of specific bZIPs at higher levels or in different tissues when a homolog is mutated. AREB3 levels are significantly increased at the shoot of *fd* mutants, possibly reducing the severity of their late-flowering phenotype (Martignago *et al.*, 2023). Thus, compensatory regulation may complicate our understanding of the contribution of individual bZIPs to particular developmental processes. In most cases, however, the regulatory role of flowering time bZIPs in flowering-unrelated molecular processes cannot be explained in terms of formation of a specific FAC. For example, FD and FDP are also involved in ABA-regulated seed germination (Romera-Branchat *et al.*, 2020), whereas stress-related bZIPs are also flowering time regulators in leaves under drought stress (Hwang *et al.*, 2019).

Independent evidence from Arabidopsis and rice demonstrates that the key florigen proteins, FT and Hd3a, respectively, regulate flowering by driving transcriptional reorganization (Taoka *et al.*, 2011; Abe *et al.*, 2019). Notably, another set of PEBPs has evolved anti-florigenic functions, antagonizing florigens at the same regulated targets (Goretti *et al.*, 2020; Zhu *et al.*, 2020). The best-characterized anti-florigenic PEBP gene in Arabidopsis is *TFL1* (Bradley *et al.*, 1997). FT and TFL1 share a high degree of sequence homology, and specific mutations are sufficient to convert FT into a TFL1 mimic (Hanzawa *et al.*, 2005; Ho and Weigel, 2014). This result strongly suggests that the mode of action of TFL1 might require the formation of a complex with FD, in a similar manner to that of the FAC. Genetic interaction studies showed that *fd* is largely epistatic to *tfl1* mutations (Hanano and Goto, 2011; Jaeger *et al.*, 2013; Cerise *et al.*, 2023). TFL1 co-localizes with FD below the meristem during vegetative development, and at the tip of the meristem after floral transition (Cerise *et al.*, 2023). This dynamic accumulation of TFL1 during development might be related to its dual regulatory role in repression of floral transition and maintenance of inflorescence meristem indeterminacy. In agreement with this, TFL1 can counteract florigen signals in the nucleus (Hanano and Goto, 2011; Goretti *et al.*, 2020; Zhu *et al.*, 2020), influencing SAM fate via short-range cell-to-cell movement (Conti and Bradley, 2007; Goretti *et al.*, 2020). A general model thus emerged where florigenic (e.g. FT) or anti-florigenic (e.g. TFL1) proteins are recruited into transcriptional complexes at target genes by the 14-3-3 protein bound to the C-terminus of a group A bZIP and, depending on which PEBPs are incorporated, different regulatory outcomes occur (Taoka *et al.*, 2013; Lifschitz *et al.*, 2014; Zhu *et al.*, 2021). Consistent with this idea, mutations in these bZIPs lead to reduced/impaired florigen/anti-florigen-mediated transcriptional reprogramming, highlighting their key role in the signaling process (Jaeger *et al.*, 2013; Cerise *et al.*, 2023).

Potato (*Solanum tuberosum*) provides an interesting organogenesis model, where flowering is induced by the FT-like florigen SELF-PRUNING 3D (StSP3D) in the shoot apex whereas tuberization is promoted by its paralog StSP6A in stolons (Navarro *et al.*, 2011). The closest FD homolog in potato, StFD-like 1 (StFDL1), is encoded by two homoeologous genes in the tetraploid potato genome and has been studied mainly in the context of tuberization. StFDL1 is expressed in roots and developing stolons (Teo *et al.*, 2017). StFDL1a and b interact with 14-3-3 proteins through their SAP motif and form a FAC-like complex called the tuberization activation complex (TAC) with StSP6A. RNAi StFDL1-impaired plants show delayed tuberization. Another FD homolog, StFD, is mainly expressed in stems and it is probably not involved in this process (Teo *et al.*, 2017). However, as seen in flowering in Arabidopsis, other potato group A bZIPs act redundantly with StFDL1. The ABI5 potato homolog StABI5-like 1 (StABL1) can bind StSP3D and StSP6A via 14-3-3 to promote flowering and tuberization (Jing *et al.*, 2022). StABL1 is probably a GA/ABA integrator in both pathways (Sun *et al.*, 2024), while StABL2, carrying an R-S/T-X-T/S-G-P C-terminal motif like Arabidopsis ABF1–ABF4, has not been characterized yet (Jing *et al.*, 2022). A recent review (Mathura *et al.*, 2024) covers in detail the molecular and morphological aspects of tuberization. In onion, FT homologs control bulb formation, but the involvement of 14-3-3, bZIPs, or a FAC-like complex has only been proposed (Lee *et al.*, 2013).

In summary, despite the evolutionary conservation and empirical validation across various plant species, significant gaps persist in our understanding of the absolute necessity of bZIPs in all aspects of florigen signaling, their role in mediating diverse signals, and their specificity in recognizing different DNA motifs.

## How do bZIP TFs and PEBPs interact?

FD-like proteins (subgroup IV) from various species require phosphorylation at a specific residue in their SAP motif for proper function (Fig. 1B; Table 1). Genetic, molecular, and biochemical studies have demonstrated the significance of the SAP motif in facilitating the formation of higher order complexes with PEBPs and 14-3-3 proteins (Table 1). In Arabidopsis, the PEBP family can be divided into floral inducers such as FT and TSF, floral repressors such as TFL1, Arabidopsis CENTRORADIALIS (ATC), and BROTHER OF FT AND TFL1 (BFT), and seed germination regulators such as MFT (Moraes *et al.*, 2019). 14-3-3 proteins belong to a group of phosphoamino acid-binding proteins that regulate the activity of their client proteins in diverse ways, mostly by exerting chaperone-like functions (Huang *et al.*, 2022).

**Table 1.** List of experimentally determined interactions between putative FAC components

Group A bZIP	PEBP	14-3-3	Species	Method	Reference
FD	FT	–	*Arabidopsis thaliana*	Y2H, *in vitro* pull-down, BiFC	Abe *et al.* (2005)
FD, FDP	FT, TFL1	–		Y2H	
FD, FDP	FT, TFL1	–	*Arabidopsis thaliana*	Y2H	Wigge *et al.* (2005)
FD, FDP	FT, TSF	–	*Arabidopsis thaliana*	Y2H	Jang *et al.* (2009)
ABF3	–	GRF4	*Arabidopsis thaliana*	*In vitro* pull-down	Sirichandra *et al.* (2010)
OsFD1	Hd3a	GF14b	*Oryza sativa*	Y2H, *in vitro* pull-down, EMSA, BiFC	Taoka *et al.* (2011)
FD, FDP	FT, TFL1	–	*Arabidopsis thaliana*	BiFC	Hanano and Goto (2011)
OsFD1, OsFD2, OsFD3	Hd3a	GF14b	*Oryza sativa*	Y2H, BiFC	Tsuji *et al.* (2013)
FD	FT, BFT	–	*Arabidopsis thaliana*	Y2H, BiFC, *in vitro* pull-down	Ryu *et al.* (2014)
FD	–	GRF3, GRF4	*Arabidopsis thaliana*	Y2H	Kawamoto *et al.* (2015)
OsFD1	Hd3a, RFT1, RCN1, RCN2	–	*Oryza sativa*	Y2H	Jang *et al.* (2017)
OsFD3	–	GF14c	*Oryza sativa*	Y2H	Brambilla *et al.* (2017)
OsFD1	RCN3	GF14b	*Oryza sativa*	BiFC, *in vitro* pull-down	Kaneko-Suzuki *et al.* (2018)
FD	FT	–	*Arabidopsis thaliana*	Improved BiFC	Abe *et al.* (2019)
FD	FT, TFL1	GRF7	*Arabidopsis thaliana*	EMSA	Collani *et al.* (2019)
OsFD1	Hd3a, RFT1	GF14a, GF14b, GF14c, GF14d, GF14e, GF14f	*Oryza sativa*	Y2H	Cerise *et al.* (2021)
OsFD3	Hd3a, RFT1	–		Y2H	
OsFD4	RFT1	GF14a, GF14b, GF14c, GF14d, GF14e, GF14f		Y2H, BiFC	
OsbZIP23, OsbZIP66/TRAB1, OsbZIP72	OsMFT2	–	*Oryza sativa*	Y2H, *in vitro* pull-down, BiFC	Song *et al.* (2020)
ABI5/DPBF1	TFL1	–	*Arabidopsis thaliana*	*In vivo* Co-IP, *in vitro* pull-down	Zhang *et al.* (2020)
OsbZIP66/TRAB1	OsMFT1	–	*Oryza sativa*	*In vivo* Co-IP, Y2H, *in vitro* pull-down, BiFC	Chen *et al.* (2021)
OsFD7	Hd3a, RFT1, OsFTL1	GF14b, GF14c, GF14d	*Oryza sativa*	Y2H, *in vitro* pull-down, FLIM-FRET	Kaur *et al.* (2021)
OsFD1	RFT1	GF14c	*Oryza sativa*	*In vitro* pull-down	Peng *et al.* (2021)
OsABI5/OREB1	OsMFT2	GF14h	*Oryza sativa*	Y2H, BiFC, *in vivo* Co-IP	Yoshida *et al.* (2022)
OsbZIP66/TRAB1	–	GF14h		Y2H	
OsFD2	–	GF14f	*Oryza sativa*	Y2H, BiFC	He *et al.* (2022)
All group A bZIPs	FT, TFL1	–	*Arabidopsis thaliana*	Y2H	Martignago *et al.* (2023)
FD, AREB3/DPBF3	FT, TFL1	–	*Arabidopsis thaliana*	Y2H, tobacco Co-IP, BiFC	

Among these interactions, the FT–14-3-3–FD complex is the most extensively characterized. This interaction occurs in the corpus region of the SAM, as shown by improved bimolecular fluorescence complementation (BiFC) using transgenic Arabidopsis plants expressing *FT* from a heat-shock promoter (Abe *et al.*, 2019). Yeast two-hybrid (Y2H) assays and EMSAs suggested that a conserved phosphoamino acid site of FD (T282) in the SAP motif mediates the FD–FT interaction (Abe *et al*., 2005; Wigge *et al*., 2005). Two SAM-expressed calcium-dependent protein kinases (CDPKs), CPK6 and CPK33, were proposed to phosphorylate FD at T282 (Kawamoto *et al.*, 2015). In agreement, a non-phosphorylatable version of the FD protein (fd^T282A^, threonine-to-alanine substitution) did not rescue the late-flowering phenotype of *fd* mutants (Collani *et al*., 2019). A study in rice using several *in vitro* and *in planta* assays shed light on the mechanistic basis of the importance of phosphorylation for the interaction between FD and FT (Taoka *et al.*, 2011). Non-phosphorylatable SAP motif versions of OsFD1 (Osfd1^S192A^) failed to interact with Hd3a and with GF14c, a 14-3-3 protein. Notably, heterologous assays indicated widespread interactions between 14-3-3s and several OsFD-like TFs as well as other group A bZIPs (Table 1; Tsuji *et al.*, 2013; Cerise *et al.*, 2021; Kaur *et al.*, 2021; He *et al.*, 2022; Yoshida *et al.*, 2022). Protein crystallization of the complete 14-3-3 protein, a truncated Hd3a protein lacking its C-terminus, and nine amino acids of the C-terminus of OsFD1 revealed a hexameric complex that was named FAC (Taoka *et al.*, 2011). In this model, two Hd3a monomers bind to the C-terminus of dimeric GF14c, forming two positively charged pockets to which two OsFD1 TFs phosphorylated at their C-terminus bind (Fig. 2A). The FAC exerts its function in the nucleus, despite the results of tobacco BiFC assays indicating that the initial interaction between Hd3a and 14-3-3 might occur in the cytoplasm. In tomato, mutant alleles of an FD-like gene disrupting the C4-encoded domain failed to stably retain the FAC in the nucleus, as these FD-like mutated proteins could not interact with 14-3-3s (Park *et al.*, 2014). However, *in vitro* experiments showed that Arabidopsis FD and rice FD-like proteins can interact with florigen proteins independently of 14-3-3 proteins (Abe *et al.*, 2005; Brambilla *et al.*, 2017). Similarly, fd^T282A^ can be immunoprecipitated with FT in tobacco assays (Martignago *et al.*, 2023). The discrepancies between these experiments may be explained by their heterologous nature. Nevertheless, FAC activity depends on FD-like proteins for the recognition of DNA-binding sites, and it is the recruitment of FT-like proteins that results in the activation of gene transcription.

FT and TFL1 have been proposed to antagonistically regulate floral transition and meristem determinacy of the SAM. A Y2H screen in tomato was the first study to identify interactions between FD-like and TFL1-like proteins (Pnueli *et al.*, 2001). Similarly, Arabidopsis FD and FDP interacted with TFL1 in Y2H and tobacco BiFC experiments (Abe *et al.*, 2005; Wigge *et al.*, 2005; Jang *et al.*, 2009; Hanano and Goto, 2011; Martignago *et al.*, 2023). It was hypothesized that the dual role of FD as an activator or repressor of gene expression is related to its interaction with FT or TFL1, respectively (Jaeger *et al.*, 2013; Ho and Weigel, 2014). ChIP-seq assays demonstrated that the TFL1–FD interaction is required for TFL1 recruitment to DNA, and that expression of *FT* from a steroid-inducible promoter resulted in the encoded FT competing with TFL1 from FD for binding to common target loci (Zhu *et al.*, 2020). A similar mechanism has been proposed for the regulation of flowering under high salinity, in which BFT delays flowering by competing with FT for FD binding to regulate the expression of *AP1* (Yoo *et al.*, 2010; Ryu *et al.*, 2014). In rice, the TFL1-like proteins RICE CENTRORADIALIS (RCN) were shown to antagonize florigen activity and to regulate inflorescence development by competing with Hd3a for 14-3-3 binding, thereby leading to gene repression (Kaneko-Suzuki *et al.*, 2018). The RCN–14-3-3–OsFD1 interaction was named florigen repression complex (FRC), and the balance between FAC and FRC formation is believed to be a general mechanism regulating plant reproductive development. In agreement with this, the formation of similar flowering activation or repression complexes has been observed in many species (Lifschitz *et al.*, 2014). Moreover, the occurrence of natural genetic variation in the florigen pathway resulted in advantageous traits during crop domestication (Eshed and Lippman, 2019). However, the *in vivo* formation of such complexes, particularly in their native expression domain, remains to be demonstrated.

All Arabidopsis group A bZIP TFs have the potential to interact with FT and TFL1 based on Y2H assays (Martignago *et al.*, 2023). The remarkable conservation of PEBP binding ability across structurally similar but evolutionarily divergent bZIPs suggests the existence of a shared, yet adaptable, bZIP–PEBP molecular framework that may be involved in regulating diverse biological processes. TFL1 interacts *in vivo* and stabilizes ABI5 (Zhang *et al.*, 2020). A SAP-related motif is present in the C-terminus of ABI5 (Fig. 1), which could mediate the interaction with TFL1 in a similar manner to the FRC. However, it is unclear whether this interaction is facilitated by 14-3-3 proteins or by ABI5 phosphorylation. In rice, interaction studies [Y2H, BiFC, and co-immunoprecipitation (Co-IP)] supported a model in which the transcriptional activity of OsABI5 depends on the recruitment of GF14h–OsMFT2 to the nucleus (Yoshida *et al.*, 2022). Non-phosphorylatable SAP motif versions of OsABI5 (Osabi5^S385A^) weakened these protein–protein interactions and the relocation of the complex to the nucleus. OsbZIP66 was shown to interact *in vivo* with OsMFT1 in rice leaves, with this complex regulating DNA binding affinity on drought-related genes and thereby enhancing drought resistance (Chen *et al.*, 2021). FAC-like complexes appear to be conserved in other monocots: in barley, four subgroup I bZIP TFs named HvABF1, HvABF2, HvABF3, and the seed-specific HvABI5 interacted with barley 14-3-3 proteins in Y2H and *in vitro* far-western assays. SAP mutated/truncated versions of HvABI5 showed reduced *trans*-activation activity on a synthetic promoter (Schoonheim *et al.*, 2007).

## bZIP–DNA interactions

The bZIP TFs bind to DNA as homo- or heterodimers (Fig. 2), providing enormous regulatory flexibility in target site selection. The N-terminal half of the bZIP domain contains an α-helix that contacts DNA, whereas its C-terminal half is responsible for dimerization and contains periodic repetitions of leucine residues that form a parallel coiled-coil structure—hence the leucine zipper name (Landschulz *et al.*, 1988). The leucine zipper is formed by a repeated series of seven amino acids (heptad) forming a helical turn, with the leucine usually in the fourth position of the heptad (Moitra *et al.*, 1997). The dimerization specificity is determined by the composition of this heptad (Deppmann *et al.*, 2004). In animal systems, bZIP heterodimers target DNA-binding motifs that are not bound by either of the interacting partners (Rodríguez-Martínez *et al.*, 2017). In Arabidopsis, the effects of heterodimerization in altering DNA binding profiles have been extensively studied for group C/S1 using *in vitro* techniques (Mair *et al.*, 2015; Pedrotti *et al.*, 2018; Li *et al.*, 2023), but the epigenetic and biological context in which the heterodimers might form and thus bind to DNA is not currently addressed by these techniques. Despite the potential role of homo- versus heterodimerization in affecting DNA recognition properties and repressive/activating regulatory functions, little information is available for group A bZIPs in plants.

Heterodimerization of group A bZIPs was mainly explored using *in vitro* assays or heterologous systems. In a Y2H assay, subgroup I ABF1 and ABF3 could heterodimerize with ABF1–ABF4, and ABF1 also bound to ABI5 and DPBF2 (Lynch *et al.*, 2012). EMSAs using ABA-regulated DNA sequences showed that ABI5, AREB3, and EEL can all heterodimerize (Bensmihen *et al.*, 2002; Kim *et al.*, 2002), while no DNA binding was found for the ABI5–DPBF2 heterodimer (Kim *et al.*, 2002). In the most widely accepted FAC model, OsFD1 operates as a homodimer at the plant SAM. However, only the last nine C-terminal amino acids, comprising the SAP motif, were used for crystal structure analysis, with no direct information about the leucine zipper motif (Taoka *et al.*, 2011). OsFD1 and subgroup II AREB3/EEL homologs OsbZIP42/HBF1 and OsbZIP9/HBF2 co-localize during development and can interact with PEBPs and 14-3-3 proteins, but heterodimerization among these bZIPs was not detected by Y2H assays (Brambilla *et al.*, 2017). Y2H and BiFC assays showed that OsFD1 cannot heterodimerize with OsbZIP24–OsFD3 and OsbZIP69–OsFD4 (Brambilla *et al.*, 2017; Cerise *et al.*, 2021). However, DNA affinity purification-sequencing (DAP-seq) of OsFD1 and OsFD4 identified hundreds of putative targets shared by both bZIPs, in addition to many independently bound genes (Cerise *et al.*, 2021). Despite sharing identical core DNA-binding sites, the spacing between tandem motifs was different for each TF, which suggests distinct and specific binding syntaxes. Moreover, OsFD4 and OsFD3 can form homo- and heterodimers (Brambilla *et al.*, 2017; Cerise *et al.*, 2021), and their combinatorial arrangement might define different modes of gene regulation. Similarly, OsbZIP65–OsFD7 homodimerization was detected using different assays (Kaur *et al.*, 2021). It is possible that all Arabidopsis group A bZIPs can homodimerize, even if DNA binding might be required to stabilize the dimeric structure due to a relatively short leucine zipper domain (Deppmann *et al.*, 2004). Group A bZIPs can also heterodimerize *in vivo*, and many of the group A bZIPs have redundant functions and partly overlapping expression domains. For instance, FD, FDP, and AREB3 are all expressed at the SAM during floral transition (Romera-Branchat *et al.*, 2020; Martignago *et al.*, 2023). ChIP-seq analyses found FD and FDP sharing several DNA-binding sites, which might indicate some degree of heterodimerization, but genetic interactions suggested otherwise (Romera-Branchat *et al.*, 2020). In tomato, DAP-seq of SSP, the ancestral SSP2, and the domesticated SSP2 showed that SSP and the ancestral SSP2 share most of their putative target genes, while the domesticated variant is impaired in its ability to bind these targets (Glaus *et al.*, 2025). Phylogenetic analyses indicate that paralogs of *SSP* and *FD* appeared independently after the divergence of the *Solanaceae* and *Brassicaceae* lineages, with the domesticated *SSP2* impairing the genetic redundancy between *SSP* and *SSP2*. Thus, despite the growing information about the genetic interaction of group A bZIPs in plants, the effect on DNA binding specificity and the molecular significance of heterodimerization are currently unclear. Applying methods such as sequential DAP-seq or double DAP-seq, which maps heterodimer binding sites on endogenous genomic DNA (Lai *et al.*, 2020; Li *et al.*, 2023), would help resolve these issues.

ChIP-seq studies (utilizing GFP antibodies targeting different GFP–bZIP fusion proteins) confirm an over-representation of G-box (CACGTG) and ABRE (ACGTGT/GC) motifs at the *in vivo* binding sites of FD/FDP and the ABF clade TFs, respectively (Song *et al.*, 2016; Collani *et al.*, 2019; Romera-Branchat *et al.*, 2020). Interestingly, Assay for Transposase-Accessible Chromatin with sequencing (ATAC-seq) revealed an over-representation for ABF1–ABF4- and ABI5-binding sites in ABA-induced accessible chromatin regions (ACRs) (Seller and Schroeder, 2023). In guard cells, *abf1–abf4* quadruple mutants were strongly impaired in ABA-induced chromatin opening, and the corresponding ABF-regulated ACRs were strongly enriched for ABREs. These ABA-regulated ACRs were also preferentially associated with ABA-induced transcriptional activation of neighboring genes, suggesting that the ABFs can act as strong determinants of ABA-triggered chromatin opening and gene transcriptional activation. This information is highly relevant to the broader question of how bZIPs can select their DNA-binding sites based on genomic context, as suggested by the analysis/modeling of DAP-seq and gene expression datasets (O’Malley *et al.*, 2016; Ezer *et al.*, 2017), and by the comparison of binding preferences *in vivo* and *in vitro* (Song *et al.*, 2016). Therefore, it would be intriguing to explore further whether ABFs function as pioneer TFs by binding to their DNA target sequences located in inaccessible chromatin regions (Seller and Schroeder, 2023), and to determine if they require accessory proteins to perform this role. In this context, PEBPs are unlikely to influence DNA binding selectivity, although they could confer stability of the bZIP–DNA complex (Kaneko-Suzuki *et al.*, 2018; Collani *et al.*, 2019).

## Perspectives

The subsets of group A bZIPs involved in ABA-mediated responses or floral induction have largely been studied independently, and emphasis has been placed on different aspects of their function. Nevertheless, they contain conserved domains along their entire length, and recent work has identified parallels in their regulation and activity. For example, the PEBPs FT and TFL1 were originally identified as interacting with the group A bZIPs FD and FDP at the C4 region and being floral regulators, but more recently TFL1 and the related PEBP MFT were also found to associate with the ABA-regulated bZIP ABI5 during seed development. These results together with the conservation of the C4 region pose the question of whether all group A bZIPs form transcriptional complexes related to the FAC, comprising two molecules each of the bZIP, the 14-3-3, and the PEBP. If all group A bZIPs form such complexes, the biochemical functions of the 14-3-3 and the PEBP within each of them remain to be fully understood as they have been variously described to affect protein stability or transcriptional activation ability of different group A bZIPs. In particular, the basis of specificity between members of the group A bZIP and PEBP families is unclear. Similarly, the biological distinction between ABA group A bZIPs and those involved in flowering at the shoot apex (FD and FDP) has also been weakened by the observation that AREB3, a classical ABA bZIP, is expressed at the shoot apex and can partially compensate for FD activity in *fd* mutants. This result clearly demonstrates shared functions across subgroups. Another unexplored feature of these TFs is the extent to which heterodimerization can occur. All bZIPs bind DNA in a dimeric structure, and heterodimerization across groups can create novel biological specificities. In the future, whether the formation of heterodimers across the group A subgroups represents a way in which ABA signaling and floral induction are integrated should be explored.

The binding sites for 14-3-3 and PEBPs in the C4 domain as well as the bZIP domain are located at the C-terminus of all group A bZIPs, but the functions of their long N-terminal region are less clear. In the ABA bZIPs, the N-terminus is phosphorylated in response to ABA, and this was shown to allow transcriptional activation. However, FD and FDP lack these phosphorylation sites, and in their case recruitment of FT into the FAC was proposed to be required to activate transcription. This distinction in the mechanisms underlying transcriptional activation ability highlights the need for a fuller understanding of the functions of the N-termini of all of these proteins, and for an improved understanding of the biochemical functions of the PEBPs. The mechanisms of florigen signaling and of ABA perception are two long-standing questions in plant biology that have converged on the group A bZIPs, and future studies on this fascinating family will be likely to reveal further important commonalities and differences in these pathways.
